# Valproic acid sensitizes resveratrol to induce glioma cell apoptosis via the ROS-PDCD4-Bax signaling axis

**DOI:** 10.3389/fcell.2026.1886066

**Published:** 2026-07-27

**Authors:** He Wang, Xunjuan Zhang, Jing Li

**Affiliations:** 1 Department of Neurology, Jilin Provincial People’s Hospital, Changchun, Jilin, China; 2 Neurobiology Laboratory, Wannan Medical College, Wuhu, Anhui, China

**Keywords:** apoptosis, glioma, PDCD4, reactive oxygen species, resveratrol, valproic acid

## Abstract

**Background:**

Glioma is an aggressive malignant brain tumor with poor prognosis. Resveratrol (RES) possesses anti-tumor activity yet limited clinical value. Valproic acid (VPA), a histone deacetylase inhibitor, boosts multiple anti-cancer drugs’ efficacy. This study explored RES/VPA synergy in glioma cell lines and syngeneic mouse tumors, and confirmed the reactive oxygen species (ROS)-programmed cell death protein 4 (PDCD4)-Bax cascade drives combined pro-apoptotic and anti-invasive effects.

**Methods:**

CCK-8 detected cell proliferation; morphology and AO/EB staining assessed apoptosis. Wound healing and transwell assays measured migration. Western blot analyzed apoptosis and migration-related proteins, including PDCD4. Dihydroethidium staining tested intracellular ROS. Rescue assays used N-acetylcysteine (NAC) and PDCD4 knockdown; subcutaneous syngeneic mice validated *in vivo* anti-tumor activity.

**Results:**

VPA alone exerted no significant cytotoxicity on glioma cells (p > 0.05 vs. NC), while RES inhibited U87MG and U251 cell proliferation in a concentration-dependent manner (p < 0.05, p < 0.01 vs. NC). RES/VPA co-treatment significantly enhanced cytotoxicity, suppressed migration and induced glioma cell apoptosis, with anti-tumor efficacy comparable to clinical doxorubicin (p > 0.05 Com vs. DOX; p < 0.01 Com vs. RES). Mechanistically, the combination markedly increased intracellular ROS accumulation (p < 0.001 Com vs. RES), upregulated PDCD4 expression (p < 0.01 Com vs. RES), and modulated apoptosis- and EMT-related protein levels to trigger cell apoptosis. NAC-mediated ROS scavenging or PDCD4 knockdown significantly abolished the synergistic pro-apoptotic effect of the combination therapy (p < 0.01 vs. Com), verifying the critical ROS-PDCD4 signaling function. *In vivo*, RES/VPA co-treatment significantly inhibited tumor growth (p < 0.01 Com vs. RES), and low-dose combination achieved equivalent efficacy to high-dose RES monotherapy (p > 0.05) without obvious toxic effects.

**Conclusion:**

RES combined with VPA exerts synergistic anti-glioma effects via the ROS-PDCD4-Bax signaling pathway, providing experimental evidence for VPA as an adjuvant in RES-based therapy. This combinatorial strategy overcomes the insufficient anti-tumor potency of RES monotherapy at the cellular and murine tumor level, expands the preclinical antitumor research value of natural polyphenols, and provides laboratory experimental reference for subsequent translational exploration of glioma therapy.

## 1 Introduction

Glioma is the most prevalent primary malignant tumor of the central nervous system, with its development closely associated with a background of chronic neurological disorders. Globally, glioma constitutes a major contributor to cancer-associated mortality, with persistently elevated annual incidence and mortality rates worldwide (Gisina et al., 2022; Ji et al., 2022; Zeng et al., 2022). Glioma possesses complicated proliferative, migratory and immune regulatory networks, and multiple ion channels and non-coding RNAs have been verified as critical therapeutic targets for brain malignancies (Gisina et al., 2022; Ji et al., 2022; Zeng et al., 2022). Advanced computational pathology approaches further facilitate the discovery of novel glioma molecular markers and therapeutic candidates, providing technical support for developing combinatorial small-molecule regimens (Jiang et al., 2024). Besides synthetic targeted agents, abundant plant-derived natural compounds have displayed potent anti-glioma activity by triggering mitochondrial oxidative stress and various regulated cell death modalities (Tang et al., 2026). Given the unsatisfactory single-drug efficacy of most natural polyphenols, synergistic combination strategies become urgently needed to improve therapeutic outcomes against glioma.

In the early stages, patients often exhibit nonspecific or subtle symptoms, leading to delayed diagnosis at advanced disease stages. This diagnostic delay contributes to therapeutic challenges and poor prognosis. Even with aggressive interventions such as complete surgical resection or radiotherapy, tumor recurrence remains frequent. For patients with advanced or metastatic glioma, conventional systemic therapies offer limited efficacy (De Carli et al., 2017), characterized by low objective response rates, frequent acquisition of drug resistance, and severe adverse side effects-all critical limiting factors that drastically reduce patients’ quality of life and long-term overall survival. Consequently, the 5-year overall survival rate remains unacceptably low (Li et al., 2017). Therefore, there is an urgent need to develop novel therapeutic agents with improved efficacy and reduced toxicity, as well as to explore innovative and effective combination treatment strategies.

Traditional Chinese medicine has demonstrated considerable potential in the field of anti-tumor therapy. With ongoing advances in modern pharmacology, the anticancer mechanisms of numerous traditional Chinese medicinal agents and their bioactive constituents are being progressively elucidated. Resveratrol (RES), a natural polyphenolic compound derived from grapes, berries, and other traditional dietary sources, has a long history of use. As a widely recognized natural compound, RES exhibits broad and significant biological activities, particularly in the context of antitumor effects (Brockmueller et al., 2021), and has been extensively investigated in various models of solid and hematological malignancies. RES exerts its anticancer effects through a multi-target, multi-pathway mechanism: it potently inhibits tumor cell proliferation and clonogenic capacity, induces cell cycle arrest, and robustly promotes apoptosis and autophagic cell death (Wu et al., 2019; Fan et al., 2018). Furthermore, RES suppresses tumor cell migration and angiogenesis by downregulating key molecular mediators such as matrix metalloproteinases and vascular endothelial growth factors (Lu and Chen, 2021; Xiao et al., 2024). It can also reverse multidrug resistance in tumor cells by modulating drug efflux transporters such as P-glycoprotein (P-gp) or by influencing apoptotic resistance pathways (Liping et al., 2022), while simultaneously regulating immune responses within the tumor microenvironment (Shan et al., 2023), including macrophage polarization and T cell activity.

Although RES demonstrates promising potential in clinical anti-tumor therapy, its application still faces several critical challenges that require urgent attention. First, the oral bioavailability of RES is generally low (Szymkowiak et al., 2025), primarily due to poor intestinal absorption, extensive hepatic first-pass metabolism, and P-gp-mediated drug efflux. These pharmacokinetic limitations hinder RES from achieving effective therapeutic concentrations in target tissues-such as glioma-thereby compromising its anti-tumor efficacy. Second, while RES exhibits a relatively favorable safety profile at conventional doses used for metabolic disorders, its potential toxicity warrants careful monitoring, particularly regarding possible hepatic accumulation. Notably, certain studies have indicated that excessively high concentrations of RES may induce cellular stress in normal hepatocytes. Finally, the therapeutic efficacy of RES as a monotherapy is inherently limited: some glioma cells exhibit insufficient sensitivity to RES, and adaptive resistance may develop during prolonged treatment. To fully harness its therapeutic potential and overcome these limitations, the development of synergistic combination therapies with improved efficacy and reduced toxicity has become increasingly imperative.

Valproic acid (VPA), a well-established histone deacetylase (HDAC) inhibitor (Kumar et al., 2019), has been clinically utilized for decades in the treatment of epilepsy and mood disorders, accumulating a substantial body of safety data. This extensive clinical experience provides a robust foundation for its repurposing research in oncology. The primary anti-tumor mechanism of VPA involves the specific inhibition of histone deacetylase activity, leading to a marked increase in the acetylation levels of both histone and non-histone proteins within cells (Vatankhah et al., 2024). Elevated histone acetylation promotes chromatin relaxation, thereby facilitating the reactivation of transcriptionally silenced tumor suppressor genes. Concurrently, acetylation of non-histone proteins modulates protein stability, subcellular localization, and functional activity, effectively reshaping the epigenetic landscape of tumor cells. Through these epigenetic regulatory effects, VPA can reverse aberrant gene expression patterns characteristic of cancer cells, eliciting multiple anti-cancer responses including induction of differentiation arrest, cell cycle arrest, apoptosis, and cellular senescence while also enhancing the sensitivity of tumor cells to radiotherapy, chemotherapy, and other targeted therapeutic agents (Sajadpoor et al., 2018). It is noteworthy that, across various cancer models, VPA has demonstrated significant synergistic effects when used in combination with multiple anti-tumor agents, including chemotherapeutic drugs, targeted therapies, and natural products (Li et al., 2019). For example, studies have indicated that VPA can enhance the apoptosis-inducing efficacy of sorafenib in glioma cells and potentiate the cytotoxic effects of chemotherapeutic agents such as doxorubicin (DOX). The underlying mechanisms of this synergy extend beyond epigenetic reprogramming and may also involve the modulation of the apoptotic threshold, suppression of DNA repair enzyme activity, and regulation of key signaling pathways. These findings provide a robust theoretical foundation and promising prospects for the combined use of RES and VPA in glioma therapy.

Despite the multi-target anti-glioma activity of RES, its monotherapy efficacy is limited by poor tissue bioavailability and insufficient oxidative stress induction in glioma cells. VPA, a clinically approved HDAC inhibitor, represents a rational sensitizing partner to overcome these limitations through multiple complementary mechanisms. First, VPA-mediated histone hyperacetylation relaxes compact chromatin, relieving epigenetic silencing of PDCD4-a critical pro-apoptotic tumor suppressor downstream of reactive oxygen species (ROS) signaling, thereby amplifying the transcriptional response triggered by RES-derived reactive oxygen species. Second, VPA interferes with intracellular antioxidant systems including thioredoxin and glutathione metabolism, eliminating the self-protective antioxidant buffer of glioma cells and exacerbating RES-induced ROS overaccumulation to initiate irreversible mitochondrial apoptosis. Third, while RES only moderately represses epithelial-mesenchymal transition (EMT) transcription factors Snail and Slug, VPA exerts epigenetic inhibition on these mesenchymal drivers, jointly blocking glioma migration and invasion. Fourth, VPA epigenetically suppresses the expression of anti-apoptotic Bcl-2 protein, lowering the intrinsic apoptotic threshold of tumor cells and rendering them more vulnerable to RES-mediated cytotoxicity. Collectively, the epigenetic and redox regulatory crosstalk between RES and VPA creates a synergistic anti-tumor cascade, providing a solid theoretical basis for their combined administration in glioma intervention.

ROS exhibit a double-edged sword nature in the regulation of cell fate (Zhao et al., 2023). Within physiological levels, ROS function as critical signaling molecules and actively participate in essential biological processes such as cell proliferation, differentiation, and immune responses. However, excessive accumulation of ROS leads to oxidative stress, which can damage key cellular macromolecules, including lipids, proteins, and DNA. Although tumor cells often exist in a state of persistent oxidative stress (Yan et al., 2023), they typically upregulate their antioxidant defense systems-such as glutathione, superoxide dismutase, and catalase-to counteract this condition. Nevertheless, compared to normal cells, tumor cells are more vulnerable to further elevations in ROS levels. When ROS accumulation surpasses the antioxidant capacity threshold of tumor cells, an irreversible cell death program is triggered. This includes classical pathways such as apoptosis, autophagy, and necrosis, as well as ferroptosis-a recently intensively studied form of regulated cell death that is iron-dependent and driven by lipid peroxidation.

A substantial body of research has demonstrated that RES exerts a pivotal role in its anti-tumor effects by disrupting redox homeostasis within cancer cells (Mahjabeen et al., 2022). One key mechanism underlying the anti-tumor activity of RES involves its ability to promote the generation of ROS through multiple pathways. On one hand, RES inhibits the activity of mitochondrial respiratory chain complex I, leading to impaired electron transfer and increased leakage of superoxide anions. On the other hand, RES suppresses the activity of antioxidant enzymes such as thioredoxin reductase and interferes with the metabolism of reduced coenzymes, including NADPH, thereby further enhancing ROS production. Additionally, RES downregulates critical antioxidant defense systems, such as the Nrf2 signaling pathway (Alavi et al., 2021), which compromises the antioxidant capacity of tumor cells. This pro-oxidative effect results in the substantial accumulation of ROS in glial tumor cells, ultimately exceeding their cellular survival threshold and triggering the activation of multiple cell death pathways. Therefore, this study aims to thoroughly investigate whether RES induces cytotoxicity in glial tumor cells through the excessive accumulation of ROS, and to further elucidate the specific signaling events involved in this process.

Based on the above complementary epigenetic and redox regulatory interactions between RES and VPA, this work constructed a complete experimental system including *in vitro* cellular functional assays, two independent molecular rescue validations and a syngeneic subcutaneous glioma mouse model, aiming to confirm their synergistic anti-tumor phenotype and dissect the core downstream signaling cascade responsible for combinatorial cell death induction. The present study possesses clear scientific novelty and differentiates from previously published studies focusing on individual RES or VPA monotherapy for glioma. First, this is the first systematic study to verify the synergistic anti-proliferative, anti-migratory and pro-apoptotic effects of RES combined with low-toxic VPA in human glioma U87MG and U251 cell lines and *in vivo* syngeneic tumor models, establishing a novel low-toxicity combinatorial strategy to overcome the insufficient anti-tumor potency of RES single-agent therapy. Second, unlike previous independent mechanistic studies of RES or VPA, our work innovatively identifies and validates a core ROS-PDCD4-Bax signaling axis responsible for the synergistic therapeutic effect via two rigorous reverse rescue strategies, including NAC-mediated ROS scavenging and PDCD4 siRNA knockdown, which clarifies the definite molecular linkage between ROS overaccumulation, PDCD4 upregulation and glioma cell apoptosis. Third, this study newly discovers a positive feedback regulatory loop between ROS elevation and PDCD4 activation amplified by VPA-induced epigenetic modification, which deeply explains the molecular basis of VPA-mediated RES sensitization in glioma treatment. Furthermore, we confirmed that low-dose RES/VPA combination achieves an anti-glioma effect equivalent to clinical chemotherapeutic doxorubicin but avoids potential systemic toxicity, providing preclinical experimental data and theoretical support for further translational research on combined natural compound regimens against glioma.

## 2 Materials and methods

### 2.1 Cell culture

The human neuroglial tumor cell lines U87MG and U251 were purchased from American Type Culture Collection (ATCC), United States; U87MG Cat# HTB-14^TM^, U251 Cat# HTB-17^TM^. Cells were maintained in Dulbecco’s modified eagle medium (DMEM, Gibco, United States, Cat# 11965092) supplemented with 10% fetal bovine serum (FBS, Gibco, United States, Cat# 10099141C) and 1% penicillin-streptomycin mixture (100×, Gibco, United States, Cat# 15140122). Cells were incubated in a humidified atmosphere at 37 °C with 5% CO_2_. When confluence reached 80%–90%, cells were passaged using 0.25% trypsin-EDTA solution (Gibco, United States, Cat# 25200056). Medium was refreshed every 2–3 days, and cell morphology was routinely checked under phase-contrast microscopy.

### 2.2 Cell viability assay

Cell counting kit-8 (CCK-8, Dojindo, Japan, Cat# CK04) was used to test cell viability. Cells were seeded into 96-well plates at a density of 5,000 cells per well and treated with gradient concentrations of RES, VPA, or their combination. RES (purity ≥ 99%, MCE, United States, Cat# HY-16561) and VPA (purity ≥99%, MCE, United States, Cat# HY-10612) were dissolved in DMSO (Sigma-Aldrich, United States, Cat# D8418) to prepare stock solutions. After 24 h and 48 h incubation, culture medium was removed, and 10% (v/v) CCK-8 solution was added to each well. Plates were incubated at 37 °C for 30 min, then absorbance at 450 nm was measured via a Tecan Spark microplate reader. Cell viability was normalized to the NC group absorbance value. All experiments were performed in three independent biological replicates.

### 2.3 Cell morphological analysis

U87MG and U251 cells were seeded in 6-well plates at 1 × 10^5^ cells/well and cultured to 80% confluence. Four groups were set: NC (drug-free medium), RES, VPA, Com (RES + VPA). After 24 h treatment, cells were rinsed twice with PBS (Solarbio, China, Cat# P1010). Cell morphology was photographed under an Olympus CKX53 inverted microscope for comparison of cell adhesion and intracellular granular deposits between groups.

### 2.4 Cell migration experiment

Cells were cultured to full monolayer confluence in six-well plates. A sterile pipette tip scratched uniform linear wounds, and floating debris was washed off with PBS. Corresponding drug treatments were applied. Wound width was photographed at 0 h, 24 h and 48 h, and wound healing rate was calculated to quantify horizontal migration.

200 μL serum-free cell suspension (1 × 10^6^ cells/mL) was added to upper chambers of 24-well Transwell inserts (Corning, United States, Cat# 3422, 8 μm pore). Lower chambers contained complete medium with matching drugs. After total 48 h incubation, non-migrated cells on the upper membrane were wiped away with cotton swabs. Migrated cells on the lower surface were fixed with 4% paraformaldehyde (Solarbio, China, Cat# P1110) and stained with crystal violet (Solarbio, China, Cat# G1062). Migrated cell numbers from 5 random microscopic fields were counted; all migration assays were run in triplicate.

### 2.5 Detection of apoptosis

AO/EB dual fluorescence staining kit (Solarbio, China, Cat# CA1140) was used to measure apoptotic proportion. Cells received 24 h drug treatment, collected and washed with PBS, then incubated with AO/EB working solution. Samples were imaged immediately under an Olympus BX53 fluorescence microscope. Live cells showed green AO fluorescence; apoptotic cells exhibited orange-red EB signal. Apoptotic percentage was calculated from ≥5 random fields per group, with three replicate experiments.

### 2.6 Comparing the anti-tumor effects of DOX

DOX (MCE, United States, Cat# HY-15142, 2 μM) served as positive control. CCK-8 viability test, morphological observation and AO/EB apoptosis staining were carried out on NC, Com and DOX groups following the protocols above to compare anti-tumor potency.

### 2.7 ROS detection

Dihydroethidine (DHE) fluorescent probe (MCE, United States, Cat# HY-100488, 5 μM working concentration) was used to detect intracellular ROS. Cells were seeded in six-well plates and treated with RES, VPA or combined drugs for 6 h. Cells were washed with pre-warmed PBS, incubated with DHE solution at 37 °C in dark for 30 min, then rinsed again to remove excess dye. Fluorescence images were captured at a fixed 100 ms exposure time. Mean fluorescence intensity was quantified using ImageJ software across three biological replicates per group.

### 2.8 Western blotting

After drug treatment, glioma cells were lysed with RIPA lysis buffer (Solarbio, China, Cat# R0010) supplemented with 1% PMSF solution (Solarbio, China, Cat# P0100) to extract total protein. Protein concentration was determined via a BCA kit (Thermo Fisher, United States, Cat# 23227). Equal protein mass was separated by SDS-PAGE and transferred onto polyvinylidene fluoride (PVDF) membranes (Millipore, United States, Cat# IPVH00010). Membranes were blocked with 5% skim milk powder (BD, United States, Cat# 232100) to block non-specific binding, then incubated overnight at 4 °C with primary antibodies targeting PDCD4, Bax, Bcl-2, cleaved-caspase 3, cleaved-PARP, E-cadherin, vimentin, snail, slug, N-cadherin. Tubulin (CST, Cat# 2146S) was used as internal loading control. After primary incubation, membranes were incubated with HRP-conjugated anti-rabbit IgG secondary antibody (CST, United States, Cat# 7074S). Protein bands were visualized using enhanced chemiluminescence (ECL) chemiluminescence substrate (Millipore, Cat# WBLUR0500). Band gray values were quantified with ImageJ software for relative expression analysis.

### 2.9 Rescue experiment

N-acetylcysteine (NAC, MCE, United States, Cat# HY-B0215, 5 mM) was used as ROS inhibitor. Three cell groups were set: NC, Com (RES + VPA), Com + NAC. After 24 h intervention, intracellular ROS level, cell viability, apoptotic rate, and apoptosis-related protein expression were detected using the above protocols to evaluate the rescue effect of ROS elimination.

PDCD4-targeted siRNA and negative control siRNA were synthesized by GenePharma (Shanghai, China). U87MG cells were cultured to 70%–80% confluence in serum-free medium. Transfection complex containing si-PDCD4 and Lipofectamine 2000 (Invitrogen, United States, Cat# 11668019) was prepared per manufacturer’s instructions, incubated 15 min at 25 °C, then added to cell culture medium for 24 h transfection. Three experimental groups: NC, Com, si-PDCD4. Functional rescue verification was performed via CCK-8, AO/EB staining, ROS detection and Western blot.

### 2.10 *In vivo* anti-tumor experiment

Kunming mice and G422 mouse glioma cells (National Infrastructure of Cell Line Resource, Cat# TC3007) were used to construct subcutaneous syngeneic glioma model. G422 cells originate from mouse spontaneous glioma tissue and share identical MHC genetic haplotypes with Kunming mice; matched genetic background eliminates allogeneic immune rejection, which defines this as a syngeneic tumor model, distinct from human cell xenograft models requiring immunodeficient nude mice. Intact immune function of Kunming mice reproduces physiological tumor immune microenvironment for more translational preclinical data. All animal operations complied with NIH Laboratory Animal Care Guidelines and were approved by Animal Care and Use Committee of Jilin Provincial People’s Hospital. Mice were randomly divided into 4 groups: NC, RES, VPA, Com. Subcutaneous tumor was established via dorsal flank injection of G422 cells. Drug formulation: 50 mg/kg RES, 300 mg/kg VPA, or combined regimen dissolved in sterile normal saline (Solarbio, China, Cat# S8010). Intraperitoneal administration once every 3 days for continuous 14 days. Tumor volume was measured with digital calipers (Mitutoyo, Japan) at regular intervals, calculated as V = length × width^2^/2. At endpoint, mice were euthanized via intraperitoneal pentobarbital sodium (MCE, United States, Cat# HY-15928), tumor tissues were harvested for subsequent analysis. Tumor growth inhibition rate and body weight dynamics were compared among four groups.

### 2.11 Statistical analysis

All quantitative data were expressed as mean ± standard deviation (mean ± SD). All cellular experiments contained at least three independent biological replicates. Statistical analysis was performed with GraphPad Prism 9.5. Two-group comparisons used unpaired Student’s t-test; multi-group comparisons adopted one-way ANOVA with Tukey’s post-hoc test. Significance threshold: p < 0.05. Marker rules: *p < 0.05, **p < 0.01; ^#^ stands for Com vs. RES, ^##^p < 0.01. Repeated-measures one-way ANOVA was utilized to analyze longitudinal, time-dependent tumor volume and mouse body weight data obtained from sequential measurements in the *in vivo* syngeneic glioma model.

## 3 Results

### 3.1 Synergistic cytotoxic effects of RES and VPA combination therapy on glioma cells

CCK-8 assay results demonstrate that, at a specific concentration, VPA alone does not significantly affect the proliferation of U87MG (Figures 1A,B) or U251 (Figures 1C,D) cells after 24 h (Figures 1A,C) or 48 h (Figures 1B,D) of incubation (p > 0.05 vs. NC group). In contrast, RES exhibits a pronounced concentration-dependent cytotoxic effect on glioma cells (p < 0.05, p < 0.01 vs. NC group at 40 μM and above RES concentration). Notably, the combination of RES and VPA induces significantly greater cytotoxicity compared to RES treatment alone at equivalent RES concentrations (p < 0.01 vs. RES group). These findings suggest that VPA potentiates the cytotoxic activity of RES against glioma cells. Further analysis of the CCK-8 data reveals that the combination of 0.5 mM VPA and 40 μM RES produces a marked synergistic cytotoxic effect on both U87MG and U251 cells at the 24 h time point. Therefore, in subsequent experiments, the treatment duration was primarily set at 24 h, with the RES concentration fixed at 40 μM and the VPA concentration maintained at 0.5 mM.

**FIGURE:**
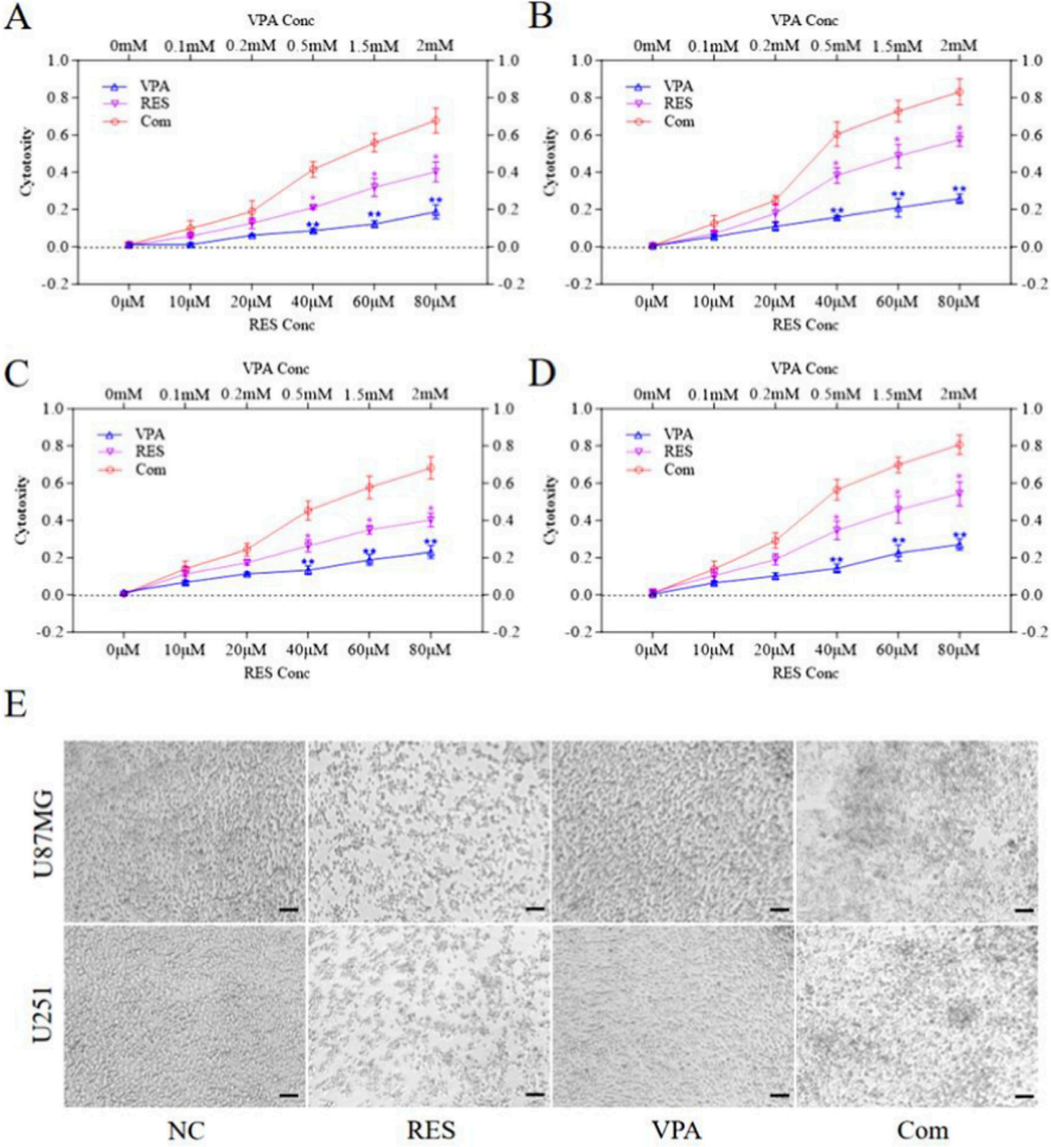
1RES and VPA exert a synergistic effect in eliminating glioma cancer cells. U87MG **(A,B)** and U251 **(C,D)** cells were treated with increasing concentrations of RES, VPA, or their combination (Com) for 24 h **(A,C)** or 48 h **(B,D)**. Cell viability was subsequently evaluated using the CCK-8 assay **(E)** Microscopic observation of the morphological changes in glioma cancer cell lines U87MG and U251 following treatment with RES (40 μM) and/or VPA (0.5 mM) for 24 h. NC: Negative control; RES: Resveratrol; VPA: Valproic acid; Com: RES + VPA. Scale bar: 100 μm. Data are shown as mean ± SD, n = 3 independent biological replicates. Statistical analysis was conducted using one-way ANOVA followed by Tukey’s post-hoc multiple comparison test. *, compared with the Com group; *: p < 0.05; **: p < 0.01.

As shown in Figure 1E, observation under an inverted bright-field microscope revealed significant differences in cell density and optical properties of U87MG and U251 glioma cells among different treatment groups. Cells in the NC and VPA groups retained normal morphological characteristics with strong adhesion and sufficient cell numbers, and no abnormal deposits were observed in the visual field (no significant morphological difference between VPA and NC group, p > 0.05 for cell viability comparison). In the RES group, partial cells presented abnormal morphology and weakened adhesion capacity, remained in a suspended state, and were accompanied by granular deposits induced by cellular damage and metabolic disorder (cell viability significantly lower than NC group, p < 0.01). The Com group exhibited a marked reduction in cell adhesion, accompanied by extensive cell suspension and detachment, as well as a sharp increase in granular deposits (cell viability significantly lower than RES group, p < 0.01). Overall, these results intuitively demonstrated that the combined treatment of RES and VPA exerted a synergistic cytotoxic effect on glioma cells, which was consistent with the aforementioned CCK-8 assay results.

### 3.2 VPA enhances the anti-migratory effect of RES in glioma cells

**FIGURE 2:**
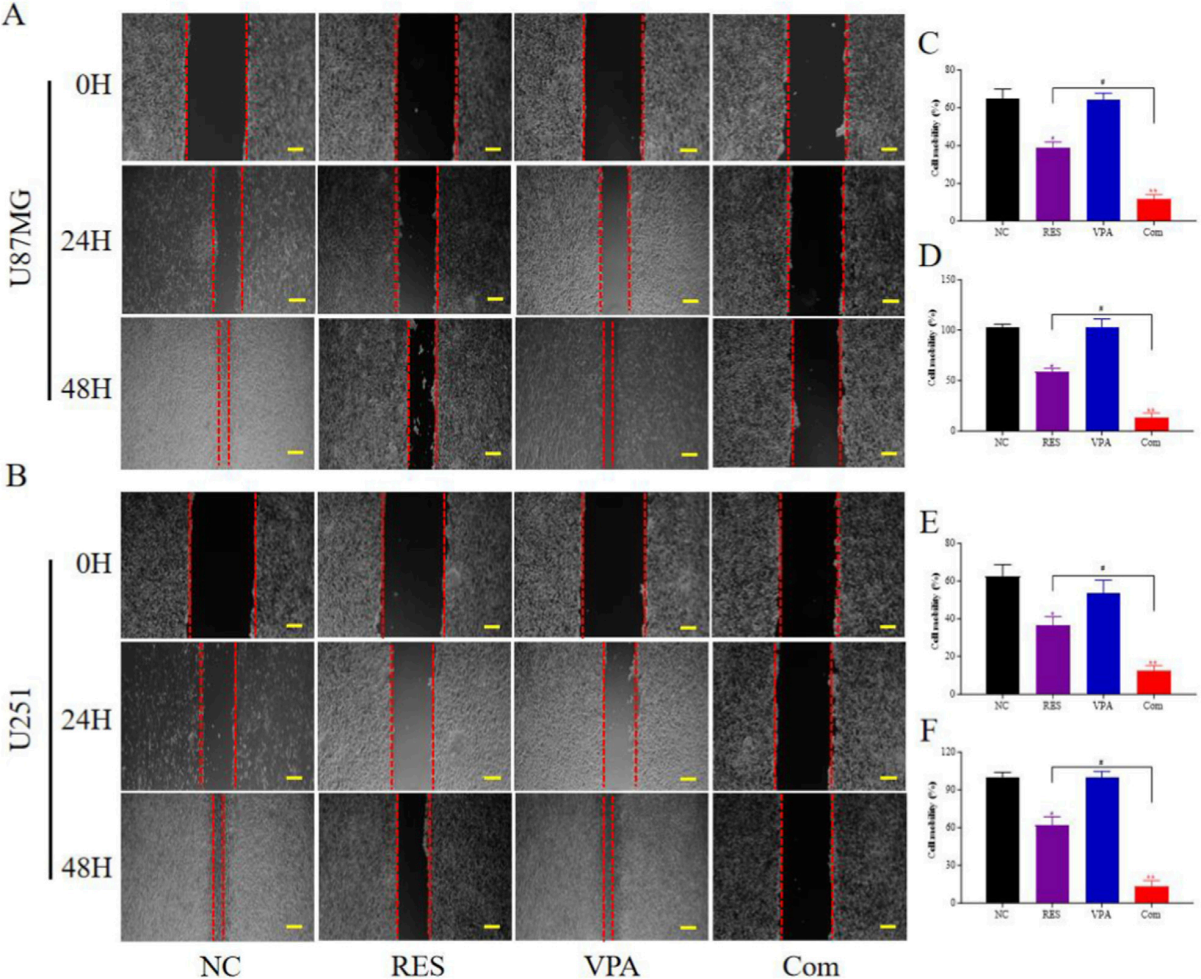
RES and VPA inhibit the migratory ability of glioma cells **(A,B)** Representative images of the scratch wound healing assay. U87MG **(A)** and U251 **(B)** cells were treated with RES (40 μM), VPA (0.5 mM), or their combination (Com), and the wound closure was photographed at 0 h, 24 h, and 48 h. Scale bar: 100 μm. **(C–F)** Quantitative analysis of the wound healing rate. The relative wound closure percentage was calculated for U87MG cells at 24 h **(C)** and 48 h **(D)**, and for U251 cells at 24 h **(E)** and 48 h **(F)**. NC: Negative control; RES: Resveratrol; VPA: Valproic acid; Com: RES + VPA. Data are shown as mean ± SD, n = 3 independent biological replicates. Statistical analysis was conducted using one-way ANOVA followed by Tukey’s post-hoc multiple comparison test. *, compared with the NC group; *: p < 0.05; **: p < 0.01; ^#^: compared with the Com group; ^#^: p < 0.05.

**FIGURE 3:**
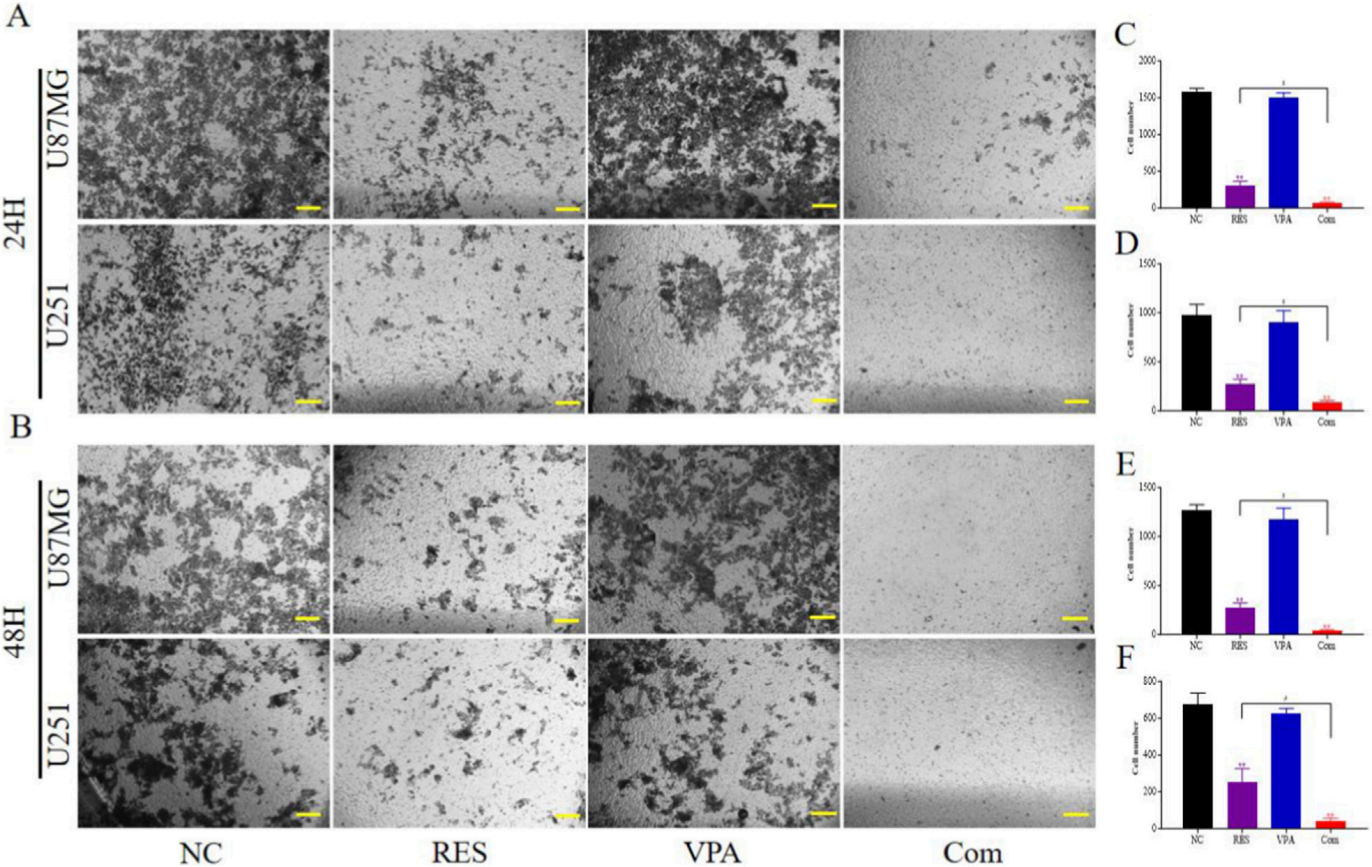
RES and VPA inhibit the migratory ability of glioma cells. **(A,B)** Representative images of the transwell migration assay. U87MG **(A)** and U251 **(B)** cells were treated with RES (40 μM), VPA (0.5 mM), or their combination (Com), and the migrated cells were stained and photographed after 24 h and 48 h of incubation. Scale bar: 50 μm. **(C–F)** Quantitative analysis of the number of migrated cells. The relative number of migrated cells was counted for U87MG cells at 24 h **(C)** and 48 h **(D)**, and for U251 cells at 24 h **(E)** and 48 h **(F)**. NC: Negative control; RES: Resveratrol; VPA: Valproic acid; Com: RES + VPA. Data are shown as mean ± SD, n = 3 independent biological replicates. Statistical analysis was conducted using one-way ANOVA followed by Tukey’s post-hoc multiple comparison test. *, compared with the NC group; *: p < 0.05; **: p < 0.01; ^#^: compared with the Com group; ^#^: p < 0.05.

**FIGURE 4:**
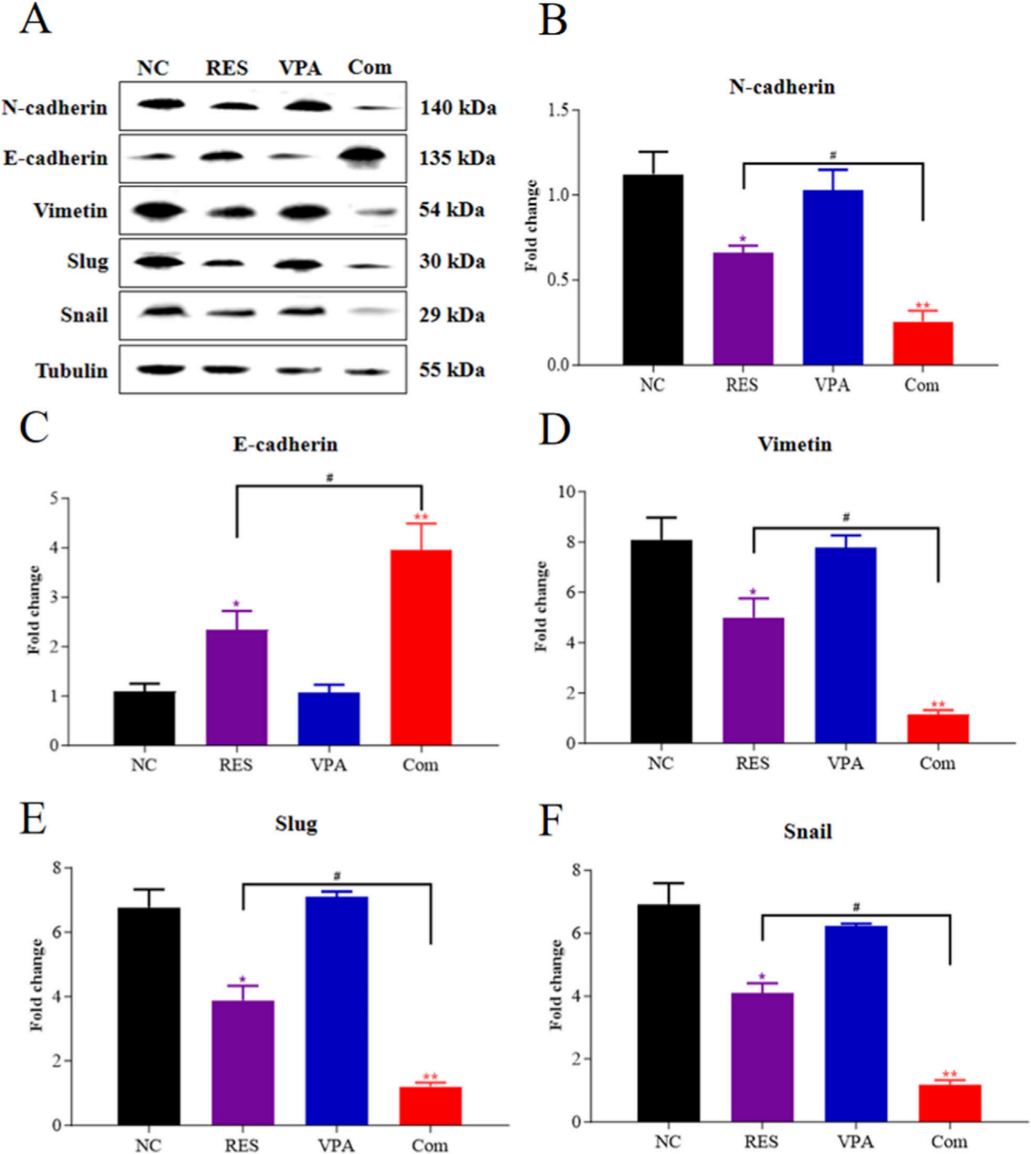
RES and VPA regulate the expression of EMT-related proteins in glioma cells. **(A)** Representative Western blot images showing the protein levels of N-cadherin, E-cadherin, vimentin, slug, and snail in glioma cells treated with RES (40 μM), VPA (0.5 mM), or their combination (Com). Tubulin served as the loading control. **(B–F)** Quantitative analysis of the relative protein expression levels of N-cadherin **(B)**, E-cadherin **(C)**, vimentin **(D)**, slug **(E)**, and snail **(F)**. NC: Negative control; RES: Resveratrol; VPA: Valproic acid; Com: RES + VPA. Data are shown as mean ± SD, n = 3 independent biological replicates. Statistical analysis was conducted using one-way ANOVA followed by Tukey’s post-hoc multiple comparison test. *, compared with the NC group; *: p < 0.05; **: p < 0.01; ^#^: compared with the Com group; ^#^: p < 0.05.

For patients with glioma, tumor metastasis significantly complicates treatment, as the metastatic process is highly dependent on the migratory capacity of cancer cells. Consequently, suppressing tumor cell migration is essential to mitigating the adverse effects associated with metastasis. In this study, cell migration activity was assessed using scratch wound healing and transwell migration assays. The results demonstrated that RES effectively reduced the rate of re-attachment of U87MG and U251 cells at the wound site (wound healing rate significantly lower than NC group at 24 h and 48 h, p < 0.05). In contrast, treatment with VPA alone had minimal impact on glioma cell migration (wound healing rate no significant difference vs. NC group, p > 0.05); however, VPA markedly enhanced the inhibitory effect of RES on cell migration (wound healing rate significantly lower than RES single group, p < 0.01, Figure 2). The findings from the transwell migration assay further corroborated these observations. Compared to the NC group, both the RES-treated group and the Com group exhibited a significant reduction in the number of cells traversing the polycarbonate membrane (p < 0.05 vs. NC group for RES; p < 0.01 vs. NC group for Com), with the Com group showing the lowest level of migration (p < 0.01 vs. RES group). In contrast, no significant difference in migratory capacity was observed between the VPA group and the NC group (p > 0.05, Figure 3). Furthermore, the expression levels of key migration-related proteins in U87MG cells were evaluated by western blot analysis. The results showed that compared with the NC group, the expression of N-cadherin, vimentin, snail and slug was significantly downregulated in the RES and Com groups (p < 0.05 vs. NC for RES; p < 0.01 vs. NC for Com), with the most prominent inhibitory effect observed in the Com group (p < 0.01 vs. RES group). Meanwhile, the expression level of E-cadherin was elevated in both the RES and Com groups (p < 0.05 vs. NC for RES; p < 0.01 vs. NC for Com), reaching the highest level in the combined treatment group (p < 0.01 vs. RES group). Notably, protein expression in the VPA group showed no significant changes relative to the NC group (all EMT protein markers p > 0.05 vs. NC group, Figure 4).

### 3.3 VPA enhances the potential of RES to induce apoptosis in glioma cells

**FIGURE 5:**
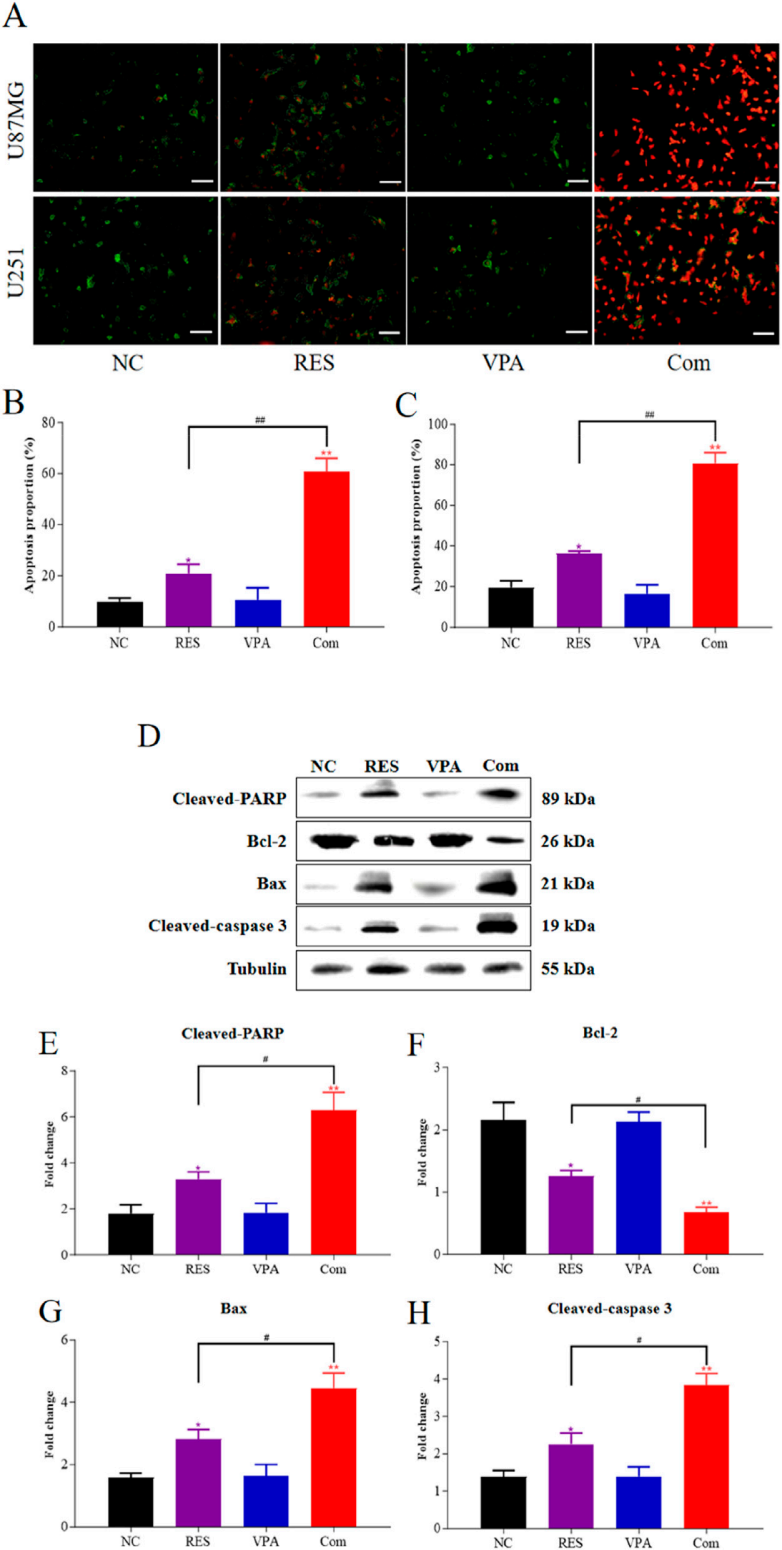
RES and VPA synergistically induce apoptosis in glioma cells. **(A)** Representative fluorescence images of U87MG and U251 cells stained with an apoptosis detection kit, following treatment with RES (40 μM), VPA (0.5 mM), or their combination (Com) for 24 h. Scale bar: 50 μm. **(B,C)** Quantitative analysis of the apoptotic rate in U87MG **(B)** and U251 **(C)** cells **(D)** Representative Western blot images showing the expression levels of apoptosis-related proteins (cleaved-PARP, Bcl-2, Bax, cleaved-caspase 3). Tubulin served as the loading control. **(E–H)** Quantitative analysis of the relative protein expression levels of cleaved-PARP **(E)**, Bcl-2 **(F)**, Bax **(G)**, and cleaved-caspase 3 **(H)**. NC: Negative control; RES: Resveratrol; VPA: Valproic acid; Com: RES + VPA. Data are shown as mean ± SD, n = 3 independent biological replicates. Statistical analysis was conducted using one-way ANOVA followed by Tukey’s post-hoc multiple comparison test. *, compared with the NC group; *: p < 0.05; **: p < 0.01; ^#^: compared with the Com group; ^#^: p < 0.05; ^##^: p < 0.01.

The apoptosis status was assessed using the AO/EB dual fluorescence staining method. Under fluorescence microscopy, viable cells exhibited green fluorescence due to AO uptake, whereas apoptotic cells displayed orange-red fluorescence as a result of EB uptake. Compared with the NC group, the number of cells exhibiting orange-red fluorescence was significantly increased in both the RES group and the Com group (apoptotic rate p < 0.05 vs. NC for RES; p < 0.01 vs. NC for Com). Notably, the Com group showed the highest number of orange-red fluorescent cells among all four groups (apoptotic rate p < 0.01 vs. RES group). No significant difference in the number of red-fluorescent cells was observed between the NC group and the VPA group (apoptotic rate p > 0.05, Figures 5A–C). These findings indicate that RES induces apoptosis in glioma cells, and at the concentration used in this study-where VPA alone exerts no significant effect-VPA potentiates the pro-apoptotic activity of RES. Western blot analysis was employed to assess the expression levels of apoptosis-related proteins in U87MG cells. Compared with the NC group, the RES group exhibited a significant upregulation in the expression of cleaved-caspase 3, Bax, and cleaved-PARP (p < 0.05 vs. NC group). In contrast, the expression of Bcl-2 was markedly downregulated in the RES group. Furthermore, the expression pattern of these apoptosis-related proteins in the Com group closely resembled that observed in the RES group, but the effects were more pronounced (cleaved-PARP, Bax, cleaved-caspase 3 p < 0.01 vs. NC; Bcl-2 p < 0.01 vs. NC; all apoptotic markers p < 0.01 vs. RES group, Figures 5D–H). These findings indicate that RES induces substantial alterations in the expression of key regulatory proteins involved in apoptosis. When compared with the RES group, the combination treatment in the Com group exerted a similar yet stronger effect on the expression of these apoptotic markers. Collectively, the experimental results support the conclusion that VPA enhances RES-induced apoptosis in glioma cells.

### 3.4 Combination therapy with RES and VPA exhibits therapeutic efficacy comparable to DOX in glioma cells

**FIGURE 6:**
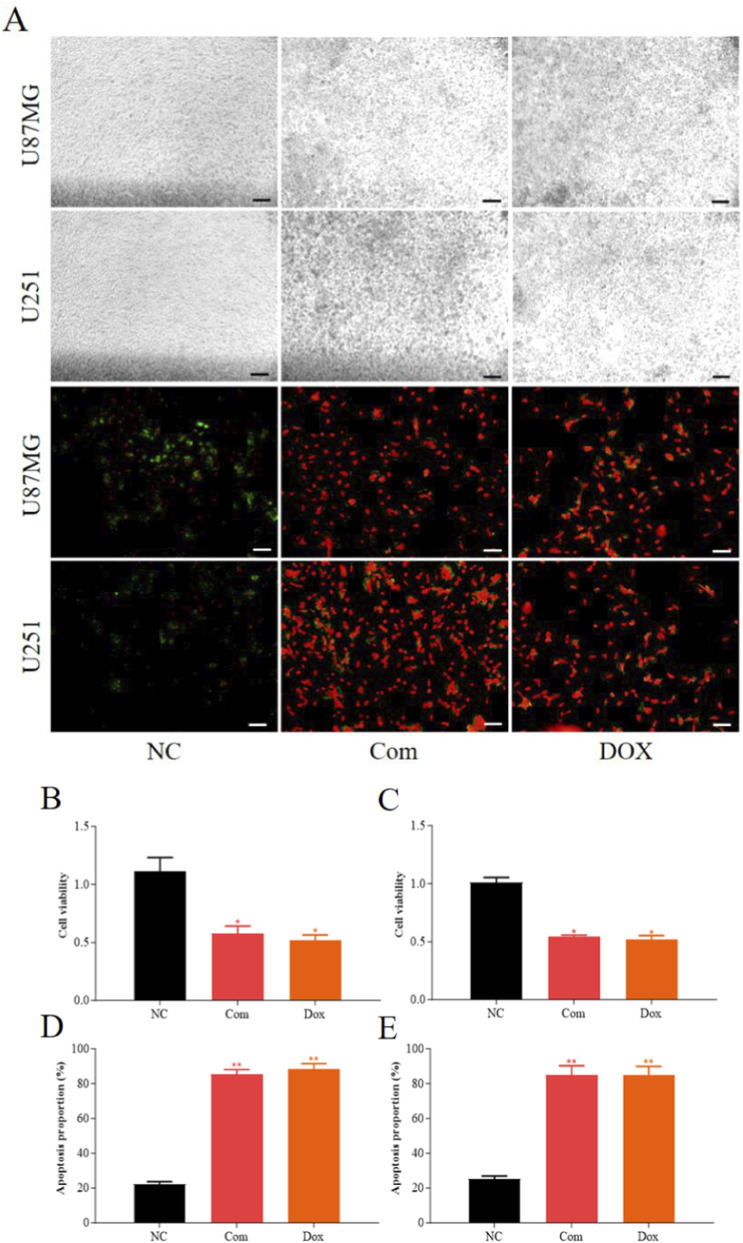
The RES-VPA combination exhibits comparable anti-tumor efficacy to DOX in glioma cells. **(A)** Representative phase-contrast images (upper panels) showing morphological changes in U87MG and U251 cells after 24 h treatment with Com (40 μM RES and 0.5 mM VPA) or DOX (2 μM). AO/EB fluorescence staining images (lower panels) visualize apoptotic cell death under the same conditions. Scale bar: 100 μm. **(B,C)** Quantitative analysis of cell viability in U87MG **(B)** and U251 **(C)** cells. **(D,E)** Quantitative analysis of the apoptotic proportion in U87MG **(D)** and U251 **(E)** cells. NC: Negative control; RES: Resveratrol; VPA: Valproic acid; Com: RES + VPA; DOX: Doxorubicin. Data are shown as mean ± SD, n = 3 independent biological replicates. Statistical analysis was conducted using one-way ANOVA followed by Tukey’s post-hoc multiple comparison test. *, compared with the NC group; *: p < 0.05; **: p < 0.01.

We assessed the cell growth of U87MG and U251 cells following incubation with Com (RES + VPA) and DOX through morphological analysis. The results revealed that, in the NC group, both cell lines exhibited tight adherence and high cellular density. In contrast, the Com group and the DOX group displayed a marked reduction in cell density and significantly weakened adhesion. Scattered particles indicative of cellular damage were observed within the field of view. Quantitative analysis demonstrated no significant difference in the inhibition of cell proliferation between the Com group and the DOX group (cell viability p > 0.05 Com vs. DOX; both p < 0.01 vs. NC group, Figures 6A–C), suggesting that the growth-inhibitory effect of the RES and VPA combination on glioma cells is comparable to that of DOX (Figures 6A–C). Similar observations were obtained in the cell apoptosis assay. Dual fluorescence staining with AO/EB showed that cells in the NC group emitted predominantly green fluorescence, indicative of viable cells. In contrast, the Com group and the DOX group exhibited a significant increase in orange-red fluorescence, representing apoptotic cells, with comparable intensities of apoptotic signals in both cell types. Further quantitative evaluation confirmed no significant difference in the proportion of apoptotic cells between the Com group and the DOX group, indicating that the pro-apoptotic efficacy of the RES and VPA combination is consistent with that of DOX (apoptotic rate p > 0.05 Com vs. DOX; both p < 0.01 vs. NC group, Figures 6A,D,E). These findings collectively demonstrate that the combined use of RES and VPA effectively suppresses the proliferation of glioma cells and induces apoptosis, with potency comparable to that of DOX, a drug commonly used in clinical practice.

### 3.5 RES and VPA combination therapy induces ROS accumulation

**FIGURE 7:**
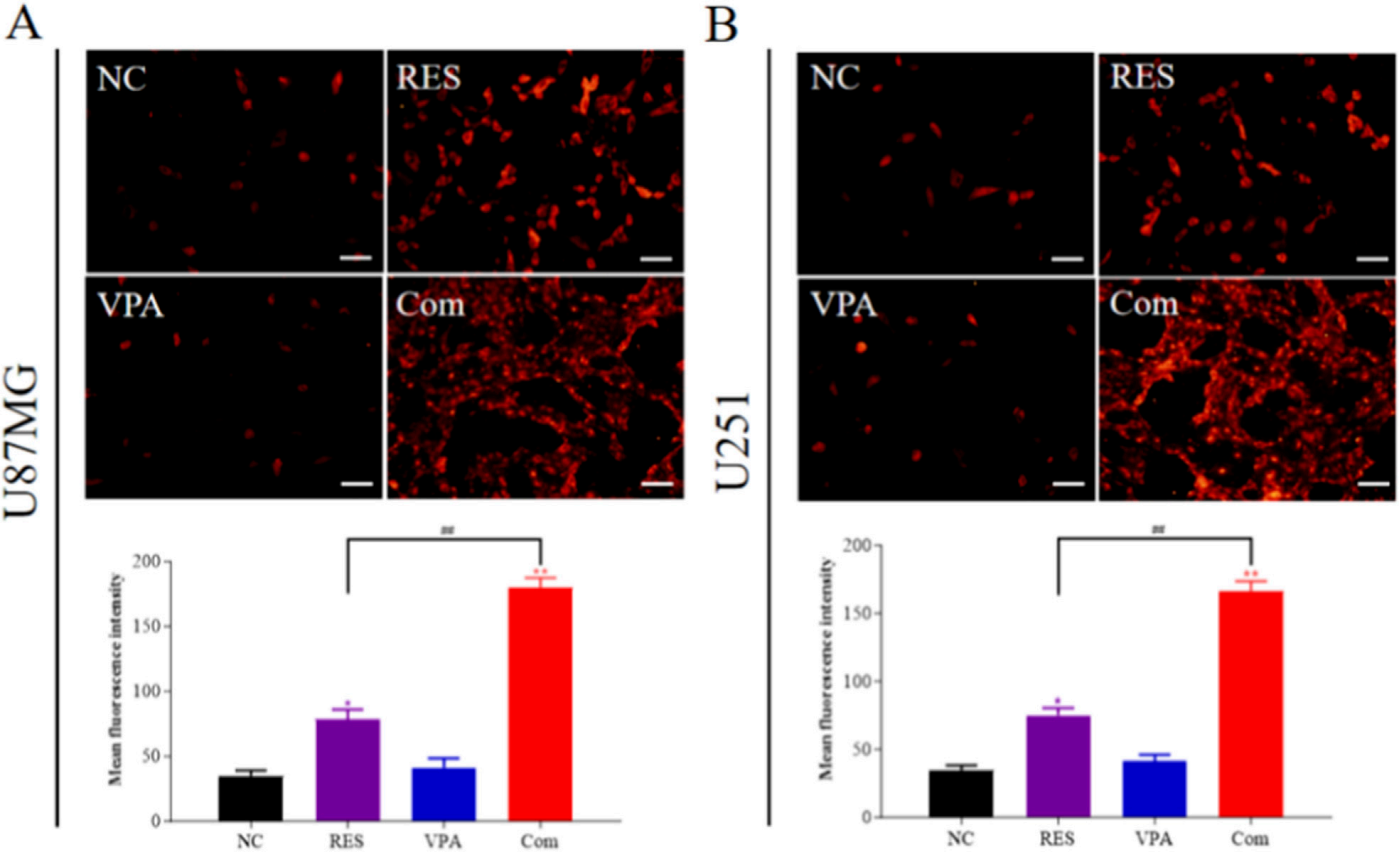
RES and VPA synergistically promote intracellular ROS accumulation in glioma cells. **(A,B)** Representative fluorescence images showing ROS accumulation in U87MG **(A)** and U251 **(B)** cells treated with RES (40 μM), VPA (0.5 mM), or their combination (Com) for 6 h. Scale bar: 50 μm. The lower panels display the quantitative analysis of mean fluorescence intensity for each group. NC: Negative control; RES: Resveratrol; VPA: Valproic acid; Com: RES + VPA. Data are shown as mean ± SD, n = 3 independent biological replicates. Statistical analysis was conducted using one-way ANOVA followed by Tukey’s post-hoc multiple comparison test. *, compared with the NC group; *: p < 0.05; **: p < 0.01; ^#^: compared with the Com group; ^#^: p < 0.05; ^##^: p < 0.01.

Previous studies have demonstrated that RES can promote intracellular ROS accumulation to exert its anti-tumor effects. To investigate whether the combination of RES and VPA could synergistically amplify this effect, we used the fluorescent probe DHE to detect ROS levels in U87MG and U251 cells, with fluorescence intensity quantified by fluorescence microscopy. In U87MG cells (Figure 7A), the NC group showed minimal basal ROS fluorescence, while the RES group exhibited a moderate increase in red fluorescence intensity, indicating a significant induction of ROS accumulation compared to the NC group (mean fluorescence intensity p < 0.05 vs. NC group). The VPA group, however, showed no significant difference in fluorescence intensity from the NC group, suggesting that VPA alone does not induce substantial ROS accumulation (p > 0.05). Notably, the combination treatment (Com) group displayed the most intense red fluorescence, which was significantly higher than that of both the RES and VPA groups (p < 0.001 vs. RES group; p < 0.01 vs. NC group), confirming a synergistic effect of RES and VPA in promoting ROS production.

Consistent results were observed in U251 cells (Figure 7B). The NC group showed low baseline ROS levels, and the VPA group did not differ significantly from the NC group (p > 0.05). The RES group showed a notable increase in fluorescence intensity (p < 0.05 vs. NC group), while the Com group exhibited the highest ROS fluorescence intensity, which was significantly elevated compared to all other groups (p < 0.001 vs. RES group; p < 0.01 vs. NC group). These results confirm that RES and VPA act synergistically to promote ROS accumulation in glioma cells, with RES serving as the primary inducer of ROS and VPA enhancing this effect when combined.

### 3.6 ROS scavenging abrogates the synergistic cytotoxicity of RES and VPA in glioma cells

**FIGURE 8:**
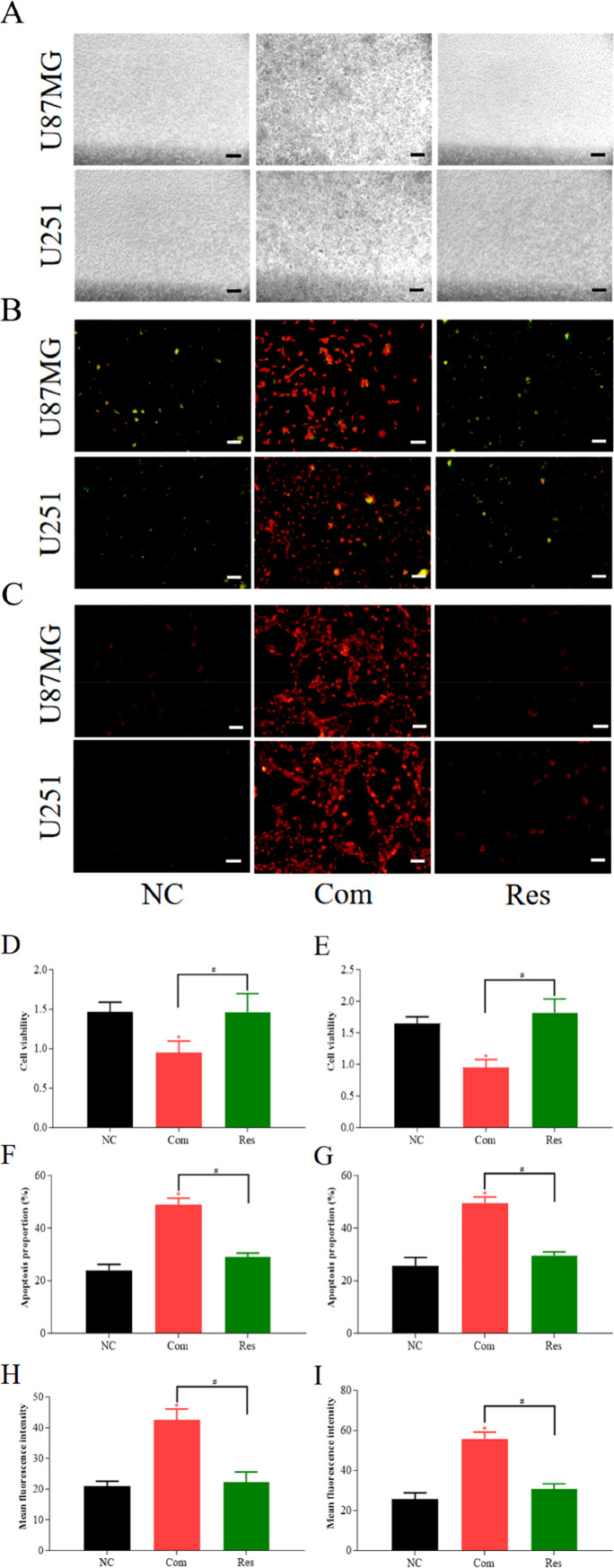
NAC reverses the anti-tumor effects of the RES-VPA combination (Com) in glioma cells. **(A)** Representative phase-contrast images showing morphological alterations in U87MG and U251 cells after 24 h treatment with NC, Com, or Com + NAC (5 mM). Scale bar: 100 μm. **(B)** AO/EB fluorescence staining images visualizing apoptotic cell death in the corresponding groups (24 h post-treatment). Scale bar: 50 μm. **(C)** DHE staining images assessing intracellular ROS accumulation in each group (6 h post-treatment). **(D,E)** Quantitative analysis of cell viability in U87MG **(D)** and U251 **(E)** cells. **(F,G)** Quantitative analysis of the apoptotic proportion in U87MG **(F)** and U251 **(G)** cells. **(H,I)** Quantitative analysis of mean fluorescence intensity (reflecting ROS levels) in U87MG **(H)** and U251 **(I)** cells. NC: Negative control; RES: Resveratrol; VPA: Valproic acid; Com: RES + VPA; NAC: N-acetylcysteine. Data are shown as mean ± SD, n = 3 independent biological replicates. Statistical analysis was conducted using one-way ANOVA followed by Tukey’s post-hoc multiple comparison test. *, compared with the NC group; *: p < 0.05; **: p < 0.01; #: compared with the Com group; ^#^: p < 0.05; ^##^: p < 0.01.

To determine whether ROS accumulation plays a critical role in inducing apoptosis in glioma cells, we employed NAC, a well-established antioxidant, to attenuate intracellular ROS levels. First, we repeated the ROS detection assay to validate the efficacy of NAC in reducing ROS. In the DHE staining experiments, treatment with RES and VPA in combination markedly enhanced ROS accumulation in both U87MG and U251 cells (Figure 8C). Quantitative analysis of DHE fluorescence intensity further confirmed that the Com group exhibited significantly higher ROS levels than the NC group (p < 0.01 vs. NC group, Figures 8H,I). Notably, the fluorescence intensity in the Res group-treated with NAC alongside RES and VPA-was substantially lower compared to the Com group (p < 0.01 vs. Com group), and nearly returned to the baseline levels of the NC group (p > 0.05 vs. NC group), confirming that NAC effectively reduces ROS levels in glioma cells.

We then captured phase-contrast microscopic images of U87MG and U251 cells treated with RES and VPA, both in the presence and absence of NAC, after 24 h of incubation. Microscopic observations revealed that cells in the NC group presented high cell density and intact intercellular connections. In comparison, cells in the Com group exhibited obvious morphological damage, including weakened cell adhesion, cell detachment from the culture surface, and the appearance of granular substances. These characteristics are closely associated with apoptosis and cytotoxic stress. Notably, cells in the RES group restored satisfactory adhesion ability, and the cell density was comparable to that of the NC group (Figure 8A). These morphological changes were further supported by quantitative CCK-8 assay, which demonstrated that the Com group exhibited a significant reduction in cell viability compared to the NC group (p < 0.01 vs. NC group, Figures 8D,E), while NAC co-treatment in the Res group significantly restored cell proliferation to near-control levels (cell viability significantly higher than Com group, p < 0.01; p > 0.05 vs. NC group). These findings collectively demonstrate that attenuation of ROS levels can reverse the cytotoxic effects induced by the combination of RES and VPA in glioma cells.

The accumulation of ROS has been shown to induce cell apoptosis (Zhang et al., 2020). To further investigate whether the induction of apoptosis is associated with ROS accumulation resulting from treatment with RES and VPA, we employed NAC as an antioxidant agent and evaluated apoptosis using AO/EB dual fluorescence staining. AO/EB staining allows for the clear distinction between live cells (green fluorescence), early apoptotic cells (green-yellow fluorescence), and late apoptotic/necrotic cells (orange-red fluorescence). Compared with the NC group, which showed minimal apoptotic cells, the Com group exhibited a marked increase in the number of early and late apoptotic cells, which was consistent with our previous findings (Figure 8B). Quantitative analysis of apoptotic rates confirmed that the Com group had a significantly higher apoptosis rate than the NC group (p < 0.01 vs. NC group, Figures 8F,G). Notably, upon co-treatment with NAC, the apoptosis rate in the Res group was significantly reduced compared to the Com group (p < 0.01 vs. Com group) and was nearly restored to the level observed in the NC group (p > 0.05 vs. NC group, Figures 8B,F,G). These results suggest that RES- and VPA-induced ROS accumulation plays a critical role in triggering apoptosis in glioma cells.

### 3.7 RES and VPA synergistically induce cell apoptosis through the ROS-PDCD4-Bax axis

**FIGURE 9:**
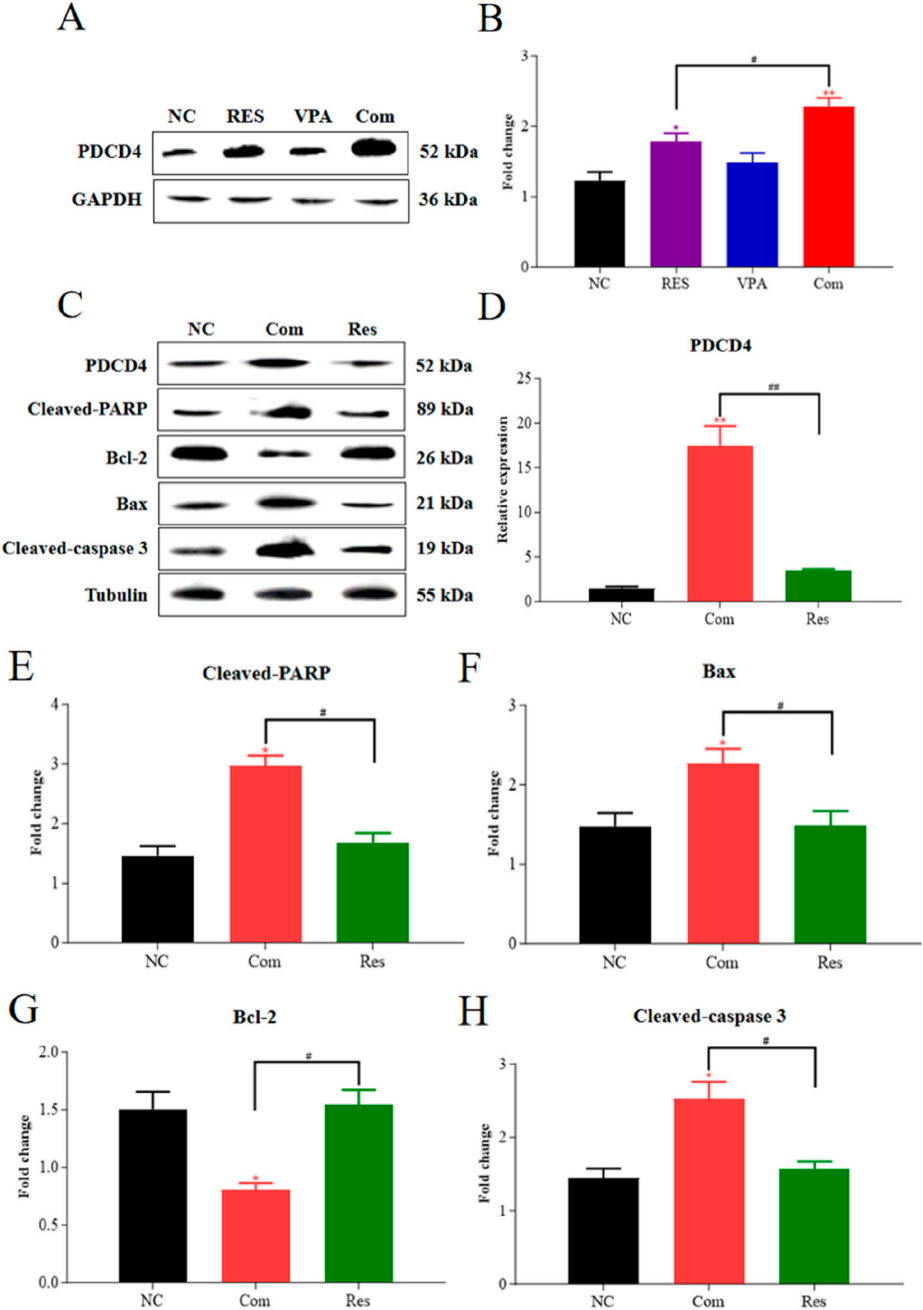
NAC reverses the RES-VPA combination (Com)-induced upregulation of PDCD4 and apoptosis in glioma cells **(A)** Representative western blot images showing PDCD4 protein expression in cells treated with NC, RES, VPA, or Com for 24 h.Tubulin served as the loading control. **(B)** Quantitative analysis of relative PDCD4 protein expression levels. **(C)** Representative Western blot images showing the expression of PDCD4 and apoptosis-related proteins (cleaved-PARP, Bax, Bcl-2, cleaved-caspase 3) in cells treated with NC, Com, or Com + NAC (labeled as Res) for 24 h. Tubulin served as the loading control. **(D–H)** Quantitative analysis of the relative protein expression levels of PDCD4 **(D)**, cleaved-PARP **(E)**, Bax **(F)**, Bcl-2 **(G)**, and cleaved-caspase 3 **(H)**. NC: Negative control; RES: Resveratrol; VPA: Valproic acid; Com: RES + VPA; NAC (Res): N-acetylcysteine (labeled as Res in the figure). Data are shown as mean ± SD, n = 3 independent biological replicates. Statistical analysis was conducted using one-way ANOVA followed by Tukey’s post-hoc multiple comparison test. *, compared with the NC group; *: p < 0.05; **: p < 0.01; #: compared with the Com group; ^#^: p < 0.05; ^##^: p < 0.01.

To further explore the mechanistic connection between ROS accumulation and apoptosis, we focused on the key regulatory gene PDCD4, which has been proposed to act as a molecular bridge between ROS accumulation and apoptotic pathways (Lu et al., 2020). Western blot analysis confirmed that RES treatment significantly upregulated PDCD4 protein expression in U87MG cells (p < 0.05 vs. NC group), with the highest expression observed in the combination treatment (Com) group (p < 0.01 vs. NC group; p < 0.01 vs. RES group), whereas no significant difference was detected between the VPA and NC groups (p > 0.05, Figure 9A). Quantitative analysis of PDCD4 protein levels further validated these observations, showing a 2.2-fold increase in the Com group compared to the NC group (Figure 9B). To verify whether the RES-VPA combination induces apoptosis through the ROS-PDCD4-Bax signaling axis, we pretreated U87MG cells with the ROS scavenger NAC and examined PDCD4 expression via western blot analysis. The results revealed that RES-VPA treatment significantly increased PDCD4 protein expression, and this upregulation was strongly inhibited by ROS scavenging with NAC (PDCD4 expression p < 0.01 vs. Com group, Figures 9C,D). This finding directly links ROS accumulation to PDCD4 upregulation, confirming that ROS is an upstream regulator of PDCD4 expression in this context.

Consistently, the expression of apoptosis-related proteins exhibited a parallel pattern to PDCD4 expression. Specifically, elevated PDCD4 expression was associated with increased Bax levels, reduced Bcl-2 expression, and higher levels of cleaved-caspase 3 and cleaved-PARP (Figures 9C,E–H). Quantitative densitometric analysis confirmed that the Com group exhibited a 2.3-fold increase in Bax protein levels (p < 0.01 vs. NC group), a 50% reduction in Bcl-2 levels (p < 0.01 vs. NC group), and a 3-fold increase in cleaved-caspase 3 and cleaved-PARP levels (p < 0.01 vs. NC group) compared to the NC group. In contrast, NAC co-treatment in the Res group significantly attenuated these changes, restoring the expression of these apoptotic markers to near-control levels (all apoptotic protein markers p < 0.01 vs. Com group; p > 0.05 vs. NC group, Figures 9E–H). These results confirm that the synergistic induction of apoptosis by RES and VPA is mediated via the ROS-PDCD4-Bax axis.

**FIGURE 10:**
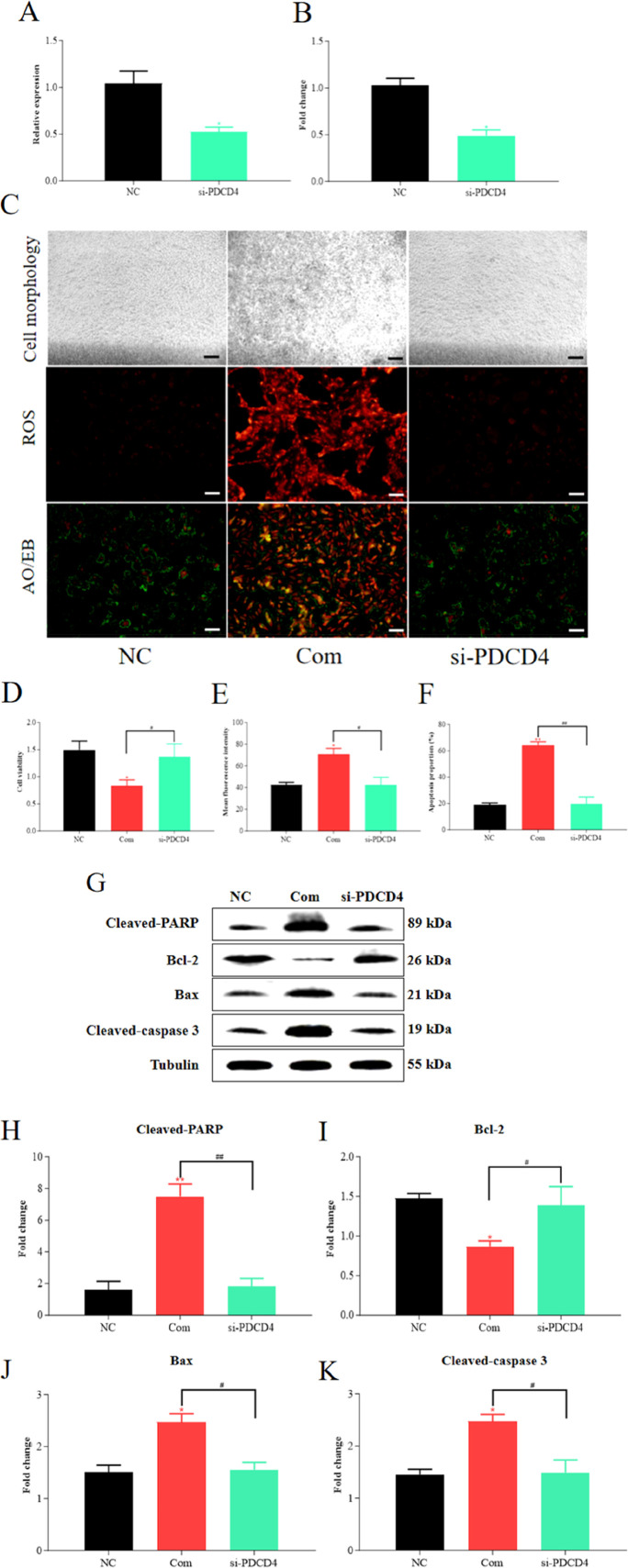
PDCD4 knockdown reverses the anti-tumor effects of the RES-VPA combination (Com) in glioma cells. **(A,B)** Verification of PDCD4 knockdown efficiency in glioma cells using siRNA, assessed by quantitative PCR **(A)** and Western blot **(B,C)** Representative images of cell morphology (phase-contrast), intracellular ROS accumulation (DHE staining), and apoptotic cell death (AO/EB staining) in NC, Com-treated, and si-PDCD4-transfected cells (24 h post-treatment). Scale bar: 100 μm (cell morphology); 50 μm (ROS, AO/EB). **(D)** Quantitative analysis of cell viability in each group. **(E)** Quantitative analysis of mean fluorescence intensity reflecting ROS levels **(F)** Quantitative analysis of the apoptotic proportion in each group **(G)** Representative Western blot images showing the expression of apoptosis-related proteins (cleaved-PARP, Bcl-2, Bax, cleaved-caspase 3) in NC, Com-treated, and si-PDCD4-transfected cells. Tubulin served as the loading control **(H–K)** Quantitative analysis of the relative protein expression levels of cleaved-PARP **(H)**, Bcl-2 **(I)**, Bax **(J)**, and cleaved-caspase 3 **(K)**. NC: Negative control; RES: Resveratrol; VPA: Valproic acid; Com: RES + VPA. Data are shown as mean ± SD, n = 3 independent biological replicates. Statistical analysis was conducted using one-way ANOVA followed by Tukey’s post-hoc multiple comparison test. *, compared with the NC group; *: p < 0.05; **: p < 0.01; #: compared with the Com group; ^#^: p < 0.05; ^##^: p < 0.01.

To further confirm that the RES-VPA combination induces apoptosis through the PDCD4-Bax pathway, we generated PDCD4-knockdown U87MG cells using siRNA. Quantitative PCR and Western blot analysis confirmed that PDCD4 expression was efficiently knocked down at both the mRNA and protein levels (PDCD4 expression p < 0.01 vs. NC group, Figures 10A,B). Functional assays demonstrated that PDCD4 knockdown significantly increased the resistance of U87MG cells to RES-VPA combination treatment. Specifically, cell proliferation assays (Figure 10D) showed that the Com group caused a marked reduction in cell viability compared to the NC group (p < 0.01 vs. NC group), while PDCD4-knockdown cells exhibited a significant recovery in proliferation capacity (cell viability p < 0.01 vs. Com group; p > 0.05 vs. NC group), confirming that PDCD4 deficiency attenuates the cytotoxic effects of the RES-VPA combination. Morphological analysis showed that, compared to control cells, PDCD4-knockdown cells maintained stronger adhesion and higher cell density after combined treatment (Figure 10C). AO/EB staining further confirmed that PDCD4 knockdown reduced the number of apoptotic cells induced by the RES-VPA combination (apoptotic rate p < 0.01 vs. Com group, Figures 10C,F). Western blot assays (Figures 10G–K) consistently demonstrated a reduced apoptotic response in PDCD4-knockdown cells, as evidenced by decreased Bax expression, increased Bcl-2 levels, and reduced cleavage of caspase 3 and PARP (all apoptotic markers p < 0.01 vs. Com group). Notably, the fluorescence intensity of the ROS probe in the si-PDCD4 group was significantly weaker than that in the Com group (ROS mean intensity p < 0.01 vs. Com group, Figures 10C,E), indicating that PDCD4 is not only a downstream target of ROS but also involved in regulating ROS accumulation, thus forming a positive feedback loop. Collectively, these findings support the existence of a positive feedback loop between PDCD4 and ROS in glioma cells, which amplifies the apoptotic response induced by the RES-VPA combination.

### 3.8 Synergistic inhibition of glioma cell proliferation by RES and VPA

**FIGURE 11:**
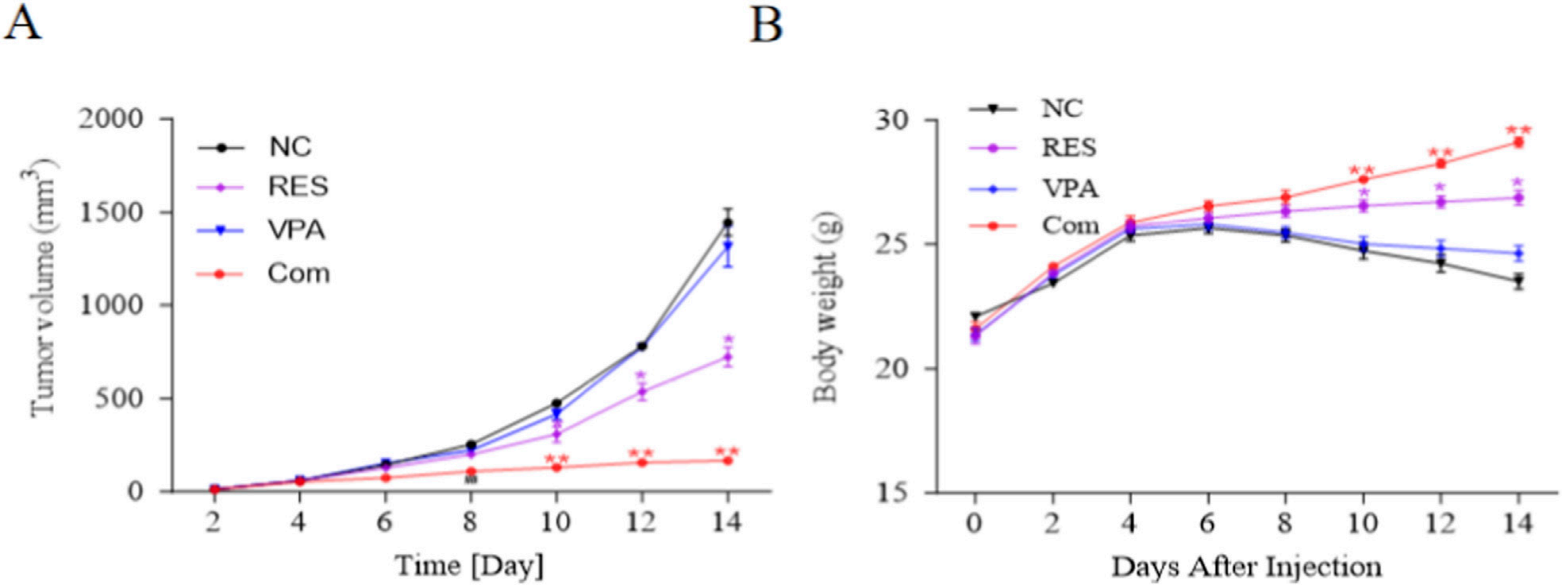
*In vivo* experimental results. **(A)** Tumor volume growth curve; **(B)** Mouse body weight change curve. NC: Negative control; RES: Resveratrol; VPA: Valproic acid; Com: RES + VPA. n = 6 mice per group. Data are presented as mean ± SD. Repeated-measures one-way ANOVA was used for statistical comparison of longitudinal tumor volume and body weight data. *, compared with the NC group; *: p < 0.05; **: p < 0.01.

*In vitro* experiments have demonstrated that the combination of RES and VPA exerts an inhibitory effect on glioma cells. To further investigate the *in vivo* antitumor efficacy of this combination, a subcutaneous syngeneic glioma mouse model was established. Mice were administered continuous intraperitoneal injections for 2 weeks prior to sacrifice, during which tumor volume and body weight were dynamically monitored. As shown in Figure 11, the combined treatment group exhibited a significantly greater tumor suppression effect compared to the RES group, the VPA group, and the NC group, with the slowest tumor volume growth rate (tumor volume p < 0.01 vs. NC group; p < 0.01 vs. RES group; VPA group tumor volume p > 0.05 vs. NC group at late time points, Figure 11A). Regarding safety, mice in the combination therapy group maintained a stable and sustained body weight gain throughout the study. This was markedly superior to the NC group, in which rapid tumor proliferation led to nutrient depletion, resulting in stagnation or even decline in body weight (body weight p < 0.01 vs. NC group at day 14). Moreover, the combination group outperformed the single-agent groups, which, despite showing certain anti-tumor activity, exhibited minor toxicity-related fluctuations in body weight (RES group body weight p < 0.05 vs. Com group at late time points; VPA group body weight p < 0.01 vs. Com group, Figure 11B). These findings indicate that the combination of RES and VPA not only enhances the inhibition of glioma growth but also mitigates the body weight loss and potential toxicities associated with monotherapy, highlighting its dual advantages of synergistic anti-tumor activity and improved safety profile.

## 4 Discussion

Glioma is among the most prevalent and aggressive malignant tumors worldwide, particularly in Asian populations, as highlighted by Kansara et al. in their 2014 study, which underscores the significant clinical severity of this disease. The onset of glioma is typically insidious, and the tumor is highly prone to both local invasion and distant metastasis. Furthermore, the development of drug resistance often limits the efficacy of systemic therapies, leaving many patients with suboptimal treatment outcomes (Tateishi, 2021). Furthermore, chemotherapy-induced adverse effects-including nausea, vomiting, bone marrow suppression, and immunosuppression (Saito, 2021)-significantly compromise patients’ quality of life. Therefore, there is an urgent need to identify effective therapeutic agents for the management of glioma.

Although previous studies have established the anti-glioma efficacy of RES (Luís et al., 2023), its clinical application has been significantly limited by toxicity and other constraints. To address these limitations, the present study investigates the repurposing of VPA, a clinically established histone deacetylase inhibitor, as a potential adjunctive therapeutic agent. By promoting chromatin relaxation, VPA is expected to enhance the sensitivity of tumor cells to treatment. This study represents the first investigation into the synergistic effects of RES and VPA in the treatment of glioma. *In vitro* experimental results demonstrated that the combination of RES and VPA exerted a significantly stronger inhibitory effect on glioma cells compared to RES treatment alone, indicating a clear synergistic interaction. RES induces glioma cell toxicity by promoting the accumulation of ROS. However, the precise molecular mechanisms through which ROS accumulation triggers apoptotic cell death are complex and warrant further in-depth investigation. Consistent with our results, multiple natural small molecules exert anti-glioma effects by elevating intracellular ROS to initiate regulated cell death. Paeonol suppresses glioma cell proliferation via ROS-dependent ferroptosis, demonstrating the central role of oxidative stress in mediating natural compound-induced tumor cell death (Li et al., 2026). In this study, through key molecular detection, western blot analysis, and a series of rigorous *in vitro* rescue experiments, we confirmed that the combined application of RES and VPA induces glioma cell apoptosis via the ROS-PDCD4-Bax signaling pathway. The findings demonstrated that the addition of VPA significantly enhances the cytotoxic effect of RES against glioma cells. Furthermore, at concentrations considered to be relatively safe, VPA markedly potentiates this cytotoxicity.

The prognosis for patients with glioma is generally poor, making the inhibition of cell migration a critical strategy in preventing tumor metastasis. To assess the effects of RES and VPA on the migratory capacity of glioma cells, we performed scratch wound healing assays and transwell migration assays. Both experimental approaches demonstrated that VPA significantly enhances the anti-migratory effect of RES. E-cadherin is a crucial epithelial marker for maintaining cell adhesion and tissue integrity, and it markedly inhibits the motility and migration of tumor cells. As core transcription factors of EMT, snail1 and slug, together with mesenchymal markers including vimentin, weaken intercellular adhesive junctions and enhance cell mobility. In contrast, aberrant upregulation of N-cadherin in tumors mediates abnormal intercellular interactions, induces EMT, and consequently promotes tumor cell migration and distant metastasis. Western blot analysis indicated that the expression levels of N-cadherin, snail1, slug and vimentin were downregulated in the treatment groups, while E-cadherin expression was upregulated. These changes were most pronounced in the Com group. These findings further validate the superior efficacy of combined RES and VPA treatment in inhibiting glioma cell migration.

Inducing cell apoptosis holds significant importance in suppressing tumor progression. Apoptosis staining results demonstrated that the apoptotic rate of U87MG cells was highest in the Com group, followed by the RES group, with both groups exhibiting higher rates than the NC group and the VPA group. A similar trend was also observed in U251 cells. Activation of the intrinsic apoptotic pathway is typically triggered by irreversible genetic damage, hypoxia, elevated cytoplasmic Ca^2+^ levels, and severe oxidative stress, among other stimuli. To further investigate the underlying mechanisms, we assessed the expression levels of key apoptotic proteins-including Bax, Bcl-2, cleaved-caspase 3, and cleaved-PARP-via Western blot analysis. The results revealed that RES treatment upregulated Bax expression while significantly downregulating Bcl-2 expression. These alterations promoted mitochondrial membrane permeabilization, leading to the release of pro-apoptotic factors and subsequent activation of downstream caspases. This cascade was evidenced by increased levels of cleaved-caspase 3 and cleaved-PARP, thereby enhancing apoptosis. Notably, VPA was found to exert a synergistic promoting effect in this process.

RES exerts anti-tumor effects by promoting the accumulation of ROS, a finding that has been substantiated in this study. ROS levels in cells across different experimental groups were measured using ROS-specific fluorescent probes. Given the relatively rapid degradation rate of ROS, we adjusted the measurement time point from 24 h to 6 h to better capture transient changes. The results demonstrated that the Com group exhibited the highest level of ROS accumulation, followed by the RES group. Both groups showed significantly higher ROS levels compared to the NC group and the VPA group. The observed trend in ROS accumulation was consistent with the pattern of cell apoptosis, suggesting a potential association between ROS elevation and the induction of glioma cell apoptosis (Zhou and Zhang, 2021). Elevated ROS levels can activate PDCD4 via the MAPK signaling pathway (Hou et al., 2013). Once activated, PDCD4 binds to the promoter region of the Bax gene, thereby upregulating Bax expression and ultimately triggering apoptotic cell death (Hou et al., 2013). Based on these findings, we propose a mechanistic model for RES combined with VPA in inducing apoptosis: ROS accumulation activates PDCD4 through the endogenous pathway, and subsequent binding of PDCD4 to the Bax gene promoter enhances Bax expression, leading to apoptosis in U87MG cells.

To verify our hypothesis, we performed a western blot analysis to evaluate the expression level of PDCD4. The initial results supported our hypothesis. To further investigate the underlying mechanism, we conducted an NAC rescue experiment, employing NAC as a ROS scavenger to reduce ROS accumulation in neural glioma cells. Subsequent ROS level detection revealed that the ROS concentration in the RES group was significantly lower than that in the Com group, confirming the efficacy of NAC in clearing ROS. The reduction in ROS levels reversed the cytotoxicity induced by the combined treatment of RES and VPA, and the apoptosis rate in the RES group was markedly lower compared to the Com group. As ROS levels decreased, PDCD4 expression also declined, leading to downregulation of Bax and subsequent alterations in downstream apoptosis-related proteins. To further validate whether the synergistic effect of RES and VPA induces cell apoptosis via the PDCD4-Bax pathway, we performed a PDCD4 silencing rescue experiment. The results demonstrated that PDCD4 downregulation indeed enhanced the resistance of U87MG cells to the combined RES and VPA treatment to some extent; however, this protective effect was not complete. Therefore, we speculate that, in addition to PDCD4, other factors may also play significant roles in mediating the relationship between ROS and cell apoptosis. Interestingly, the results of the rescue experiments revealed a positive feedback regulatory loop between ROS accumulation and the upregulation of PDCD4. By employing VPA to maintain chromatin in a more relaxed conformation, thereby increasing DNA accessibility, this epigenetic state further amplified the positive feedback mechanism. We hypothesize that this enhancement may underlie the role of VPA as a sensitizing agent in this context. It is evident that the rescue experiment, through reverse validation of this mechanism, further substantiates our hypothesis. This study represents the first investigation into the potential of combining RES and VPA for the treatment of glioma. The findings demonstrate that RES exerts a significant antitumor effect, which aligns with previously reported research. Notably, the combination of RES and VPA produces a more pronounced antitumor activity compared to RES monotherapy. Collectively, the *in vivo* experimental results indicate that the synergistic action of RES and VPA effectively suppresses tumor growth. Therefore, this combinatorial therapeutic strategy exhibits synergistic anti-glioma effects in preclinical models, which may lay a theoretical foundation for subsequent translational study of glioma intervention. However, this study has several limitations. First, the precise upstream mechanisms driving ROS accumulation remain unclear and warrant further investigation. Second, in addition to PDCD4, other signaling pathways may also contribute to ROS-mediated apoptosis and require elucidation. Furthermore, the specific molecular mechanisms underlying the anti-migratory effects observed in this study merit deeper exploration in future studies.

## 5 Conclusion

In summary, as a potent enhancer of RES, VPA significantly augmented the anti-tumor efficacy of RES *in vitro*, offering valuable insights into overcoming the current limitations associated with RES application. The combination of RES and VPA induces glioma cell apoptosis through the ROS-PDCD4-Bax signaling pathway, thereby exerting pronounced anti-tumor effects. This study not only deepens the understanding of ROS accumulation and apoptotic mechanisms in glioma at the cellular level but also underscores the pivotal role of PDCD4 in this process. Furthermore, it proposes a novel, synergistic drug-based therapeutic strategy for glioma treatment.

## Data Availability

The original contributions presented in the study are included in the article/supplementary material, further inquiries can be directed to the corresponding author.
